# Transoral Laser Resection of the Tongue Base in the Workup of Unknown Primary Head and Neck Squamous Cell Carcinoma

**DOI:** 10.7759/cureus.41103

**Published:** 2023-06-28

**Authors:** Sarah P Chan, Justin McLarty, Elizabeth Knecht, Steve C Lee

**Affiliations:** 1 Otolaryngology - Head and Neck Surgery, Loma Linda University School of Medicine, Loma Linda, USA; 2 Otolaryngology - Head and Neck Surgery, Kaiser Permanente, Riverside, USA; 3 Otolaryngology, Dayton Children's Hospital, Dayton, USA

**Keywords:** panendoscopy, head and neck cancer, unknown primary neoplasm, base of tongue, transoral laser

## Abstract

Objective

Failure to localize the primary tumor site in head and neck carcinoma of unknown origin after imaging and endoscopic evaluation leads to increased treatment-related morbidity. The use of transoral laser microsurgery to improve the detection of unknown primary carcinoma site identification is described in this article.

Methods

A retrospective cohort of 71 consecutive cases of cervical carcinoma of an unknown primary source from 2006 until 2012 from a single academic institution was analyzed. Of these, 10 patients were excluded based on our exclusion criteria. All patients underwent endoscopy with biopsies performed by fellowship-trained head and neck cancer surgeons.

Results

The primary detection rate was 76% for patients who underwent laser tongue base resection versus 34% for traditional operative examination. There were no complications or prolonged recovery times in either group. Operative time was increased by the addition of the transoral base of tongue resection by 30 minutes.

Conclusions

Laser tongue base excision offers improved sensitivity in primary site detection without a significant increase in morbidity and only a modest increase in operative time.

## Introduction

Cervical metastasis with an unknown primary site is rare and represents 2-3% of cases of head and neck cancer with a heavy male predominance [1]. Failure to identify the primary cancer site often leads to aggressive radiotherapy of the entire upper aerodigestive tract [2,3]. This radiotherapy, especially when combined with chemotherapy, is associated with significant short and long-term morbidity [4]. Compared with focused radiotherapy to a known sub-site and its borders, wide-field radiotherapy may be more fraught with extensive post-radiation complications such as mucositis, dysphagia, xerostomia, loss of taste, decreased tongue mobility, and neck fibrosis. While improved radiotherapy technology and techniques have allowed for decreased side effects, the advantages of limiting the radiation field to a smaller area encompassing the primary tumor site are clear. In addition to the avoidance of radiotherapy-related morbidity, minimization or avoidance of radiation to the pharynx may preserve radiotherapy as a treatment option in instances of cancer recurrence or associated second primary tumors.

The identification of metastatic squamous cell carcinoma (SCC) in a cervical lymph basin initiates a workup and staging protocol which varies between institutions. Ideally and most commonly, initial cervical node sampling is via transcutaneous needle biopsy. Once a pathologic diagnosis of metastatic carcinoma has been obtained, a concerted effort is made to identify a primary site and obtain accurate staging.

Diagnostic strategies have evolved to maximize the detection of the primary site. The pharynx is the most common site of an occult primary tumor in the head and neck [1]. Office physical examination followed by imaging and operative endoscopic exam with biopsy are currently the mainstays of diagnosis. Office examination includes direct visual inspection, palpation, and laryngopharyngoscopy using transnasal endoscopy or mirror exam. Usually, a head and neck primary tumor or area of suspicion will be identified at this time.

Contrast-enhanced computed tomography (CT) and/or magnetic resonance imaging (MRI) of the neck are routinely utilized to assist in staging, followed by stage-appropriate imaging of the chest. If no primary site is identified or if the presence of distant metastasis is unclear, metabolic imaging with positron emission tomography (PET) may be performed. In the setting of an unknown primary, the role of PET scanning remains controversial largely due to a large rate of false positive findings [5].

Operative inspection with biopsies is usually performed next as the final and most definitive step in primary site identification, as this provides pathologic diagnosis and verification of a primary site. If imaging and exam are unremarkable for a possible primary lesion, traditionally, random or directed cup forceps biopsies are performed at four locations including the ipsilateral hypopharynx, tonsil, tongue base, and nasopharynx. This biopsy technique has resulted in low rates of primary site identification [6].

A more recent diagnostic approach involves more aggressive tissue sampling from the oropharynx, as this represents the site most likely to harbor occult malignancy. Unilateral or bilateral tonsillectomy greatly improves diagnostic sensitivity and is now widely employed. Studies have shown that close to 50% of primaries may be found in the tonsil [7]. Koch et al. found that 10% of primaries were in the contralateral tonsil and thus recommended bilateral tonsillectomy [8]. The generalizability of these data has been debated, though. Occult malignancy occurs at a similar frequency in the lingual tonsils as in the pharyngeal tonsils, so the concept of more aggressive tissue sampling in this area is similar to tonsillectomy. Emerging data show that base of tongue (BOT) resection may be as efficacious as tonsillectomy in primary tumor identification [5].

In this study, we report on transoral CO2 laser BOT resection as a simple technique that requires minimal specialized expertise or equipment to identify a primary tumor site. The detection of previously unknown primary sites can increase the number of patients who are candidates for more directed therapy and thus reduce treatment-related morbidity.

## Materials and methods

This study is a Loma Linda University (LLU) Institutional Review Board (IRB) exempted study due to its retrospective nature. Patient charts from an LLU Cancer Center Tumor Board database search of records from 2006 to 2012 were reviewed in this study. The records included the International Classification of Diseases (ICD)-9 codes for tumors classified as the unknown primary sites of the head and neck, the base of the tongue, tonsil, nasopharyngeal, hypopharynx, and pyriform sinus. Included were adults with an unknown primary site of the head and neck - defined as biopsy-proven, metastatic SCC in a cervical node for which a primary site was not found on initial physical exam, including flexible laryngoscopy, or on initial imaging (Table 1).

**Table 1:** Pre-operative imaging work-up PET: Positron emission tomography; CT: Computed tomography

Imaging		PET	CT	PET + CT	Neither
Laser base of tongue resection		3	17	5	4
Standard operative examination		6	9	10	7

Patients in this database were treated at either Loma Linda University Medical Center or Loma Linda Veterans Affairs Healthcare System. Excluded were patients not meeting the above inclusion criteria, those with a prior history of head and neck malignancy, and primary lesions identified on subsequent imaging. In our study 10 patients were excluded based on the above criteria; three due to a history of prior SCC of the head and neck, one because the patient desired radiation treatment prior to BOT resection or standard operative management, and six were excluded because their primary was identified on imaging (five on PET and one on CT). All imaging studies performed are noted in Table 1. Full inclusion and exclusion criteria are seen in Table 2. We also noted gender, age at diagnosis, and N stage for each patient (as defined by the American Joint Commission on Cancer) in Table 3.

**Table 2:** Inclusion and exclusion criteria CT: Computed tomography; PET: Positron emission tomography

	Criteria
Inclusion criteria	Patients over the age of 18 with metastatic squamous cell carcinoma in a neck mass by needle biopsy.
Exclusion criteria	The primary site found on office evaluation with history, physical exam, and fiberoptic endoscopy of the upper aerodigestive tract. The primary site found on preoperative CT or PET scans. Pathology other than squamous cell carcinoma. The patient was not taken for an operative examination. Previous history of head and neck cancer.

**Table 3:** Clinical profile of study cohort

	Laser base of tongue resection	Standard operative examination
Number of patients	29	32
Gender		
Male	26	30
Female	3	2
Average age at diagnosis	63.3	63.8
N stage		
N1	2	4
N2a	7	11
N2b	14	10
N2c	4	4
N3	2	3

Operative technique

Patients underwent either standard operative examination with or without the addition of laser base of tongue (BOT) resection based on surgeon preference. There was a temporal shift in technique with more recent patients more likely to receive BOT resection. The standard operative examination consisted of direct laryngoscopy under general anesthesia with directed biopsies of suspicious areas and directed biopsies of the BOT mucosa using cup forceps. Bronchoscopy, esophagoscopy, nasal endoscopy, and nasopharyngoscopy were also performed, and biopsies of any suspicious sites were obtained. Tonsillectomy was then performed using the monopolar diathermy technique. This would conclude the standard operative examination.

The other method involved performing CO2 laser BOT resection in addition to bronchoscopy, esophagoscopy, nasal endoscopy, tonsillectomy, and nasopharyngoscopy. After endoscopy and tonsillectomy were performed and no primary site was identified, an operating microscope was positioned to visualize the oropharynx and a standard Crowe-Davis or McIvor tonsillectomy retractor with a flat blade or a Steiner distending laryngoscope was used to expose the oropharynx. A CO2 laser was delivered by a micromanipulator or fiber delivery system. The lingual tonsillar tissue was ipsilateral to the neck mass from the midline to the palatine tonsillectomy wound and from circumvallate papilla to vallecula was resected using CO2 laser and an operative microscope. The deep tongue musculature was not included in the specimen. This was then oriented for permanent pathology. Generally, no additional hemostasis was required, and the procedure was completed in about 30 minutes. In most cases, neck dissection was not performed at the time of operative examination as this procedure was considered to be a diagnostic as opposed to a therapeutic procedure. Most patients were observed overnight for bleeding and adequate oral intake.

## Results

Other methods for improved diagnostic sensitivity to minimize treatment-related morbidity have been sought in recent years. We present our initial results using tonsillectomy and CO2 laser BOT resection, following standard clinical and radiographic workup, for increased sensitivity in primary cancer site detection in the setting of the unknown primary.

Of the 71 patients presenting to Loma Linda University Medical Center and Loma Linda Veterans Healthcare System from 2006 to 2012, 61 were eligible for inclusion based on study criteria. Demographic information is shown in Table 3. Approximately half the patients underwent a standard operative exam and the majority of those patients were patients who presented earlier in our series. Our series has a large preponderance of male patients and most were N2 at presentation.

We noted the detection rate for an unknown primary was 76% for patients who underwent transoral CO2 laser resection of the ipsilateral BOT compared to 34% of primary sites detected by traditional operative evaluation following initial presentation (Table 4). Most of the primary sites that were located were in the base of the tongue in the patients that underwent transoral laser resection of the BOT while in the patients who underwent a standard operative exam, the most common site of the primary lesion was in the tonsil (Table 5). The margin status for all our specimens was positive as no visible tumor was seen intraoperatively and tumors identified were only identified by microscopic examination by pathology.

**Table 4:** Identification of primary site after laser base of tongue resection or standard operative examination

	Laser base of tongue resection N (%)	Standard operative examination N (%)	Statistical significance
Primary found	22 (76%)	11 (34%)	P < 0.05
Primary not found	7 (24%)	21 (66%)	
Total	29 (100%)	32 (100%)	

**Table 5:** Location of the primary site after laser base of tongue resection or standard operative examination

Anatomic site	Laser base of tongue resection	Standard operative examination
Base of tongue	13	4
Tonsil	6	6
Nasopharynx	3	0
Hypopharynx	0	1

There were no operative complications in either the laser tongue base resection or standard examination cohort. One patient had a post-operative bleed from the tonsillectomy defect that was managed conservatively and did not require operative control of hemorrhage. Patients were able to return to oral intake immediately and were discharged home immediately or one day postoperatively. The operative time for the laser BOT resection group was approximately 30 minutes more than the standard examination group.

Therapeutic interventions after identification of the primary site varied according to patient preference and our study was not powered to determine survival or morbidity differences based on the therapy delivered. However, most patients where the primary remained unknown were treated with wide-field radiation of the upper aerodigestive tract while patients with identified primaries were treated with more directed therapy.

## Discussion

Our experience with transoral laser BOT resection demonstrated improved localization of tumors. Our study cohort has a large male preponderance which is consistent with the gender distribution of the disease. We suspect that this gender skewing does not invalidate our results. The distribution of the primary sites found in our study aligns with previous studies on unknown primary head and neck squamous cell carcinoma with the vast majority being found to be oropharyngeal [6,7,9]. The majority of positive malignant biopsies in the laser BOT group were identified in the base of the tongue while the majority of tumors in the standard examination group were in the tonsil. This probably does not indicate a selection bias between the two groups but rather that the standard examination failed to identify the tumors in the BOT due to inadequate tissue sampling. The true distribution of primary sites is more likely more accurately depicted in the laser BOT resection group which sampled the entire ipsilateral BOT mucosal surface.

All of the patients in our study were evaluated with CT and/or PET. And while a careful evaluation of imaging found the primary site in 10% of our initial cohort, it is clear that neither PET nor CT is as effective as careful operative evaluation in determining the primary site of carcinoma.

It is our experience that laser tongue base resection can dramatically increase sensitivity in primary tumor identification. By detecting occult primary tumors not found by traditional work-up, treatment planning can be focused on, decreasing the morbidity associated with wide-field aerodigestive tract radiation therapy. Furthermore, if the primary tumor is resected with adequately clear margins, adjuvant treatment may be obviated altogether in the setting of pathologic N1 disease after neck dissection [10]. This also has the benefit of preserving radiotherapy as a treatment option in case of recurrence. This is important as pharyngeal recurrence in or adjacent to an irradiated field usually results in salvage surgery with significant morbidity to the patient [11].

Several recent studies have advocated resection of the base of the tongue as a method to improve primary site detection. Karni et al. report a 94% primary detection rate with laser microsurgery of the base of the tongue in a cohort of 30 patients (18 underwent transoral laser microsurgery, and 12 underwent an exam under anesthesia). That study utilized a method that is dependent on the identification of subtle changes in the mucosa on microscopic examination and focally resecting these lesions for intra-operative pathologic analysis [6]. While that method is effective in their hands, the success of that protocol is highly operator dependent and likely requires an experienced laser microsurgeon to be able to identify these areas of concern adequately. Therefore, extrapolation of their data to centers that do not perform large numbers of laser microsurgical oncologic resections is problematic.

Several studies with cohorts of 10-20 patients have reported primary detection rates ranging from 54% to 100% using robotic surgery [12-17]. The surgical strategy between these approaches and ours is the same but with different instrumentation. Surgical robots are becoming more widespread; however, robot time is often limited and there are significant costs related to disposables in robotic surgeries. The CO2 laser is widely available and there is little conflict in terms of availability and no disposable costs unless using a fiber system. With cost efficiency becoming more important in the practice of medicine, robotic surgery has distinct disadvantages compared to laser surgery.

Our data represents one of the larger cohorts of unknown primary patients treated with the base of tongue resection. Additionally, our study has a temporally separated internal control as most of the patients before 2009 were treated with a random base of tongue biopsies, and most after 2009 were treated with a laser base of tongue resection minimizing selection bias. This technique reflects a simple application of laser microsurgery directed at tumor identification by expanded tissue sampling without the need to specifically identify focal areas of concern. The ipsilateral tonsil and the entire mucosal surface of the ipsilateral tongue base from the midline to inferior tonsillectomy defect and from the circumvallate papilla to vallecula are resected empirically, and the patient is awakened. This method has the advantage of being less operator dependent and therefore widely applicable outside of institutions with extensive transoral laser microsurgery experience in finding the primary tumor but has the potential disadvantage of increased morbidity. We found no difference in recovery time or long-term morbidity between the two groups in this study.

Our paper is focused on the diagnostic workup of unknown primary of the head and neck rather than treatment, and our dataset was not powered to address differences in survival or morbidity based on treatment modality. Currently, patients with unknown primaries are treated with multimodality therapy, most commonly, resection followed by adjuvant radiation with or without chemotherapy versus primary chemoradiotherapy with or without post-therapeutic neck dissection [18]. In the largest series of patients treated with neck dissection alone, the recurrences rate for the ipsilateral neck in Nx-1 patients was 13%, and 32% for N2-3 disease [19].

Transoral laser tongue base excision is easily performed without extensive experience in transoral laser microsurgery or the need for expensive robotic equipment and results in improved sensitivity in the detection of the unknown primary site SCC without a significant increase in complications or recovery time. We report a slight increase in operative time with the application of an empiric base of tongue resection. Much like palatine tonsillectomy is now routinely performed in the diagnostic workup of unknown primaries rather than tonsil biopsies, we advocate that BOT resection should be performed rather than BOT biopsies in the diagnostic workup of unknown primaries.

## Conclusions

Transoral CO2 laser tongue base excision offers significantly improved sensitivity in primary site detection with negligible increase in morbidity and only a modest increase in operative time. It is a simple technique that requires minimal specialized expertise or equipment to aid in the identification of a primary tumor site. The detection of previously unknown primary sites can increase the number of patients who are candidates for more directed therapy and thus reduce treatment-related morbidity.
